# Early Life Factors Associated with Adult-Onset Systemic Lupus Erythematosus in Women

**DOI:** 10.3389/fimmu.2016.00103

**Published:** 2016-03-31

**Authors:** Christine G. Parks, Aimee A. D’Aloisio, Dale P. Sandler

**Affiliations:** ^1^Epidemiology Branch, National Institute of Environmental Health Sciences, Research Triangle Park, Durham, NC, USA; ^2^Social & Scientific Systems, Inc., Durham, NC, USA

**Keywords:** lifecourse epidemiology, pesticide exposure, birthweight, preterm birth, autoimmune disease

## Abstract

**Background:**

Exposure early in life can influence adult disease and immunity, but the role of early life exposures in risk of systemic lupus erythematosus (SLE) is not established.

**Methods:**

Women in a national cohort (ages 35–74) provided data on perinatal, maternal, and sociodemographic factors, longest residence to age 14, and residential farm history of at least 12 months to age 18. Cases (*N* = 124) reported SLE diagnosed age 16 years or older with use of disease modifying antirheumatic drugs. Non-cases (*N* = 50,465) did not report lupus. Odds ratios (OR) and 95% confidence intervals (CI) were estimated by logistic regression adjusting for age and race/ethnicity.

**Results:**

SLE was associated with low birthweight (data on 84 cases and 36,477 non-cases; <2,500 versus 3,000 to <3,500 g OR = 2.2; 95%CI 1.2, 3.9) and preterm birth (57 cases and 22,784 non-cases; ≥1 month early versus full-term OR = 3.4; 95%CI 1.6, 7.4). Considering longest childhood residence to age 14, SLE was associated with more frequent pesticide use (e.g., at least monthly OR = 2.3; 95%CI 1.3, 4.1). SLE was associated with having an early and extended childhood farm residence (i.e., prenatal/maternal farm exposure and longest childhood farm residence OR = 1.8; 95%CI 1.1, 3.0 versus neither). In those with a childhood-only farm residence of 12+ months, agricultural pesticide use was associated with SLE, with the strongest associations for direct personal exposures.

**Conclusion:**

The association of SLE with preterm birth is consistent with studies in other populations and with an observed association with low birthweight. The associations of SLE with childhood exposure to residential and agricultural pesticides warrant further study.

## Introduction

Systemic lupus erythematosus (SLE) is an autoimmune disease characterized by immune reactivity to multiple nuclear components and inflammation, resulting in diverse clinical features and multiple organ involvement. The causes of SLE are generally not known. Racial disparities and increased familial risk suggest a genetic predisposition. It is believed that environmental factors may contribute to the development of disease, but knowledge on specific risk factors is mostly limited to occupational exposures (1).

The developmental origins hypothesis has been proposed for many adult-onset, chronic inflammatory diseases, including SLE (2–6). Exposures during and after gestation, including nutritional, infectious, chemical, and psychosocial factors (7–11), play a critical role in shaping future immunity. A few epidemiologic studies have examined perinatal and childhood factors associated with SLE, with results including evidence of an association with preterm birth and inconsistent associations with birthweight (12, 13).

Socioeconomic factors have been associated with disease severity, inflammation, and adverse outcomes in SLE (14, 15). Although socioeconomic disparities originating from childhood have been described across a variety of chronic inflammatory diseases (16), the role of childhood socioeconomic status (SES) and related adversities in risk of SLE is not known. Other early life immune modifying exposures (e.g., infections, vitamin D) have also been considered in relation to SLE risk (17).

History of farm occupation and pesticide exposures has been associated with SLE (18–20), but the role of childhood exposures have not been specifically examined. One past study suggested an association of SLE with mixing pesticides in a farm environment (21), while another showed a dose–response association personal pesticide in women use with rheumatoid arthritis (RA) and SLE that was highest in women with a farm history (18). Women who live or work on a farm as adults may be more likely to have grown up on a farm. Therefore, it is plausible that previous studies showing SLE associations with farm-related pesticide use may reflect to some extent an association with childhood exposures.

In this study, we examine associations between adult-onset SLE in a national cohort of women and a range of perinatal and early life exposures, some of which had previously been associated with RA in this same cohort (22). Investigating the role of early life factors in risk of SLE will help us to understand what factors underlie susceptibility for developing autoimmune disease in adulthood. Along with knowledge of genetic susceptibility, understanding the developmental origins of SLE risk may help identify opportunities for prevention and mechanisms contributing to immune dysregulation.

## Materials and Methods

### Sample and Case Identification

We conducted a case–control analysis using baseline data from the NIEHS Sister Study cohort (*N* = 50,884 women ages 35–74, enrolled 2003–2009). Women were eligible if they had a sister with breast cancer, but did not have breast cancer themselves at enrollment (23). Participants were identified through self-referral following a broad advertising campaign in the U.S. and Puerto Rico. The Sister Study was approved by the Institutional Review Board of the National Institute of Environmental Health Sciences, NIH. Participants provided written informed consent.

Probable cases (*n* = 124) were classified based on a self-reported doctor’s diagnosis of SLE at age 16 or older and reported use of disease modifying antirheumatic drugs (DMARDs). Cases with missing data on medications or not reporting DMARDs (*N* = 153), reporting non-specified or discoid lupus only (*N* = 61), or diagnosis before age 16 were excluded (*n* = 6). The most common DMARDs reported by probable cases were hydroxychloroquine (90% ever and 70% currently used) and methotrexate (11% ever and 10% current). No data on disease severity or nephritis were available; however, medications often used to treat nephritis were less common (e.g., Azothioprine; 8% ever used). Five probable cases (5% of 124) also reported a diagnosis and DMARD use for RA. Non-cases (*n* = 50,465) reported no lupus. Other possible cases of autoimmune disease were not excluded from the referent, but are unlikely to influence results due to their relative rarity in the overall sample.

### Exposures and Covariates

Computer-assisted telephone interviews collected data on sociodemographic factors, childhood residential history, lifestyle factors, personal medical history, and medication use. Questions on sociodemographic factors included food insecurity (“When you were growing up, were there times your family didn’t have enough to eat?”), highest household education level when respondent was age 13, and relative household income while growing up (well off, middle income, low income, or poor). Adult sociodemographic covariates included participant age (baseline), race/ethnicity (dichotomized for this analysis as non-Hispanic White and non-White, including black, Hispanic, and other racial and ethnic groups), and education level (dichotomized for this analysis as less than a college degree, including <12 years, high school degree or equivalent, some college but no degree, or associate degree/technical degree versus college degree, including college graduate/bachelor’s degree and graduate/professional degree).

Data on the longest childhood residence to age 14 years and childhood farm residence of 12 months or longer to age 18 were collected by computer-assisted telephone questionnaire. For the longest childhood residence, participants born in the U.S. were grouped by state into: (1) census regions (West, Midwest, Northeast, South, and Puerto Rico) and (2) stratified by 40th and 35th parallels (approximate according to U.S. state). Questions about the longest childhood residence included whether the residence was a farm, had been a farm, or was near a farm, and whether pesticides were used in and around the house or garden, including frequency of use. Women who did not report a farm as longest childhood, adult, or current residence were also asked whether they had lived on any other farm for 12 or more months. A detailed questionnaire then asked about pesticide use on crops and animals, including opportunities for personal exposures (i.e., personally mixed or applied, and presence in the field when pesticides were being sprayed) for all residential farm experience 12 months or more up to age 18. Similar questions were asked for residential and agricultural pesticides based on current and longest adult residence and any farm residence 12 months or more ages 18 years and older.

Participants were also mailed family history questionnaires that included assessment of perinatal factors and were encouraged to contact their mother or other relatives to assist with as needed to provide data on maternal age at participants’ birth, prenatal, maternal, and household smoking, maternal farm residence or work on farm while pregnant, breast feeding, birthweight (pounds, ounces), and gestational age at birth. Participants unable to report exact birthweight were asked whether birthweight was <5 pounds or at least 5 pounds, and responses were converted to grams and categorized (<2,500; 2,500–3,499; 3,400–3,999; and ≥4,000 g).

Birth order was calculated from brother birthdates reported in the family history questionnaires and sister birthdates reported during the computer-assisted telephone interviews. Other perinatal factors [multiple birth, soy formula, maternal/gestational diabetes, gestational hypertension or preeclampsia, and diethylstilbestrol (DES) exposure] were not considered due to low prevalence.

### Analyses

Analyses were conducted in SAS (version 9.2, SAS Institute Inc., Cary, NC, USA). Logistic regression was used to estimate odds ratios (OR) and 95% confidence intervals (CI). Significant findings were those in which the 95%CI excluded the null (OR = 1.0), corresponding to a two-sided *p* < 0.05. Initial models were age-adjusted and secondary models also adjusted for race/ethnicity. We also evaluated associations with a childhood SES adversity score [a sum of four factors – young maternal age at birth (<20 years), low or poor relative childhood household income, maximum household education less than college, and childhood food insecurity], which was previously examined with respect to RA in this cohort (22). For significant associations, we examined possible confounding by education and conducted sensitivity analyses excluding non-Whites (a higher risk subgroup and potential effect modifier) and women over age 60 (with higher potential for errors in reporting of perinatal and childhood factors). Results were not substantially changed unless otherwise noted. Data on adult factors (education, residential pesticides, and farm residence) were used to explore potential joint effects and identify independent “childhood only” exposures.

## Results

Cases were slightly younger than non-cases and enrolled in the cohort within a median of 11 years since diagnosis (Table 1). Cases were more likely than non-cases to be of non-White race/ethnicity (including black or Hispanic) and were less likely to have graduated from college.

**Table 1:** **Characteristics of probable SLE cases and non-cases in the Sister Study**.

	Probable SLE *N* = 124	Non-cases *N* = 50,465
	
	Median years (IQR)	
Age at enrollment	54 (47, 60)	55 (60, 61)
Age at diagnosis	43 (33, 50)	N/A
Time since diagnosis	11 (3, 16)	N/A

	***N(%)***	***N(%)***

Race/ethnicity^a^		
White	86 (69)	42,269 (84)
Non-White	38 (31)	8,181 (16)
Educational attainment		
≤High school degree	16 (13)	7,725 (15)
Some college or technical degree	59 (48)	17,010 (34)
4-year college graduate	25 (20)	13,633 (27)
Professional or graduate degree	24 (19)	12,085 (24)

*^a^Non-Hispanic White and non-White (Black – 19% of cases versus 9% of non-cases, Hispanic 9% of cases versus 5% of non-cases, and other racial and ethnic groups – 3% cases and non-cases)*.

**Figure 1:**
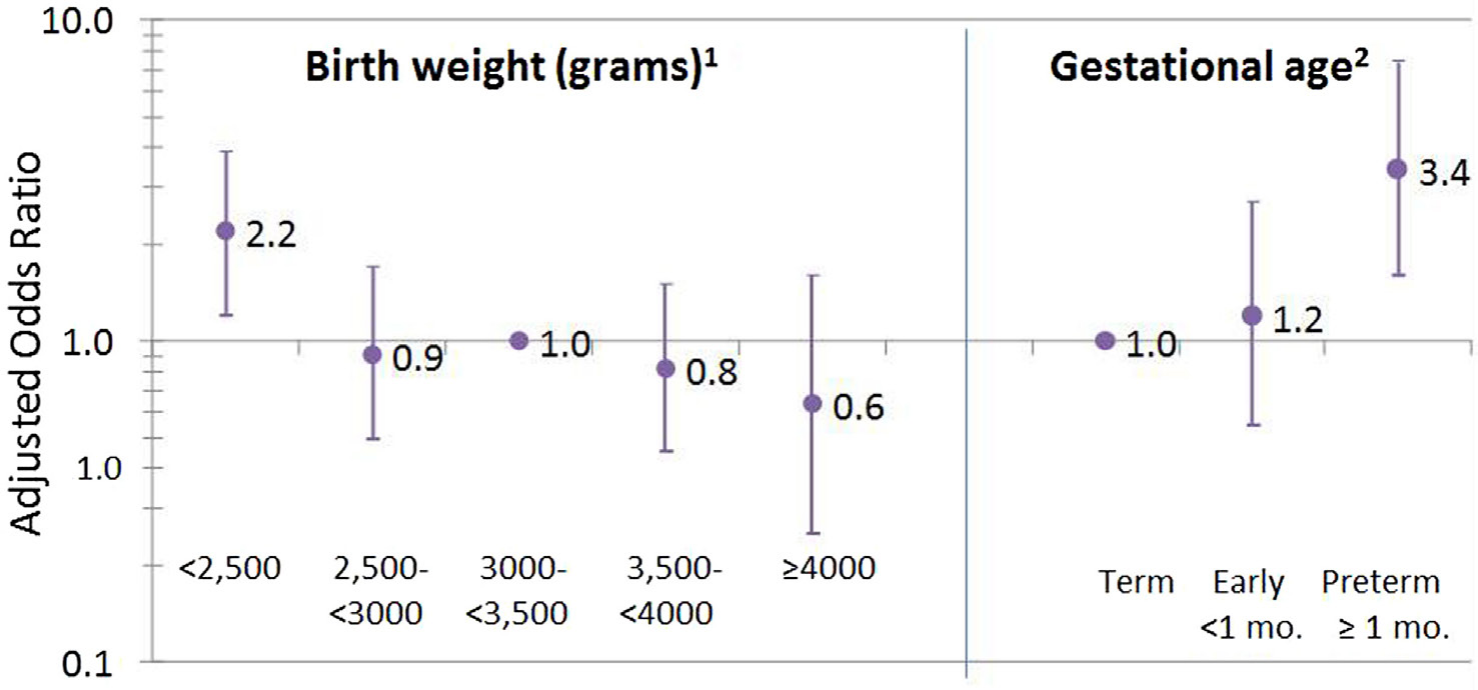
**Birthweight and gestational age associated with SLE**. ^1^Missing on 39 cases (31%) and 13,659 (27%) non-cases; test for trend across birthweight categories, *p* = 0.008. ^2^Missing or do not know for 67 cases (54%) and 27,681 (55%) non-cases.

Systemic lupus erythematosus was associated with low birthweight, with a linear dose response (*p*-trend = 0.008) and with preterm (at least 1 month early) versus term birth (Figure 1). Excluding preterm births (seven of eight preterm cases also had a low birthweight), the overall trend was no longer significant (*p* = 0.08; not shown); however, when the sample was restricted to women under age 60, the trend across low birthweight categories became significant again (*p* = 0.03, not shown). For women providing exact birthweight, cases (*N* = 84) weighed 6.8 (SD 1.6) pounds at birth compared with 7.2 (SD 1.3) in non-cases (*N* = *n* = 36,477), for an adjusted OR = 0.80; 95%CI 0.69, 0.94 per pound.

Systemic lupus erythematosus cases were significantly more likely to have been born to mothers who lived or worked on a farm while pregnant (Table 2). Birth season, prenatal smoking (maternal and household), being breastfed, firstborn, and sibling number were not significantly associated with SLE. Overall, SLE was not associated with individual childhood social factors. We saw no evidence of joint effects for combined lower adult educational attainment and increased SES score (Table S1 in Supplementary Material); in college educated women odds of SLE were almost doubled for women with two or more childhood SES adversity factors compared with only one or none (OR = 2.0; 95%CI 1.2, 3.6).

**Table 2:** **Perinatal and childhood sociodemographic risk factors for SLE**.

	Case *N* = 124	Non-case *n* = 50,465	Odds ratios^a^ (95% CI; confidence intervals)

	*N* (%)	*N* (%)	
**Birth characteristics and perinatal factors**			
Mom lived or worked on farm while pregnant			
No	88 (75)	39,560 (82)	Referent
Yes	30 (25)	8,803 (18)	1.6 (1.0, 2.4)^b^
Prenatal smoking			
None	35 (32)	15,303 (34)	Referent
Mom only	9 (8)	3,406 (8)	1.2 (0.58, 2.5)
Household only	45 (41)	14,917 (34)	1.3 (0.86, 2.1)
Both	21 (19)	10,853 (24)	0.90 (0.53, 1.6)
Birth season			
Winter (December–February)	29 (24)	12,214 (24)	1.3 (0.76, 2.2)
Spring (March–May)	32 (26)	12,157 (24)	1.4 (0.83, 2.2)
Summer (June–August)	24 (19)	13,170 (26)	Referent
Fall (September–November)	39 (31)	12,924 (26)	1.6 (0.93, 2.6)
Breastfed			
No	61 (54)	22,526 (49)	Referent
Yes	51 (46)	23,339 (51)	1.2 (0.84, 1.8)
Firstborn			
No	98 (81)	37,936 (77)	Referent
Yes	23 (19)	11,412 (23)	0.90 (0.57, 1.4)
Sibling number^c^			
One	16 (13)	6,091 (12)	1.2 (0.71, 2.2)
2 or 3	48 (40)	21,824 (44)	Referent
4 or 5	28 (23)	12,181 (25)	0.95 (0.60, 1.5)
6 or more	29 (24)	9,515 (19)	1.1 (0.70, 1.8)
**Early life social factors^d^**			
Young maternal age	11 (9)	2,358 (5)	1.7 (0.91, 3.2)
Lower income	46 (37)	17,044 (34)	1.0 (0.71, 1.5)
Lower household education	72 (60)	27,041 (54)	1.1 (0.79, 1.7)
Food insecurity	15 (12)	4,753 (12)	1.1 (0.64, 1.9)
SES adversity factors^e^			
0	34 (29)	17,643 (36)	1.0 (referent)
1	40 (34)	16,319 (34)	1.2 (0.78, 2.0)
2	33 (29)	10,817 (22)	1.4 (0.90, 7.3)
3–4	11 (9)	3,613 (7)	1.2 (0.61, 2.5)

*^a^Odds ratios and 95% confidence intervals adjusted for age and race/ethnicity*.

*^b^*p*-Value <0.05*.

*^c^Women must have a sister to be enrolled in study. Thus, all have at least one sibling*.

*^d^Young maternal age of <20 years of age at birth, relative household income reported as low income or poor, and maximum household education in adults/parents less than a college degree*.

*^e^Socioeconomic status (SES) summary score based across four variables – young maternal age, lower household income and education, and food insecurity; *p*-trend = 0.26*.

For longest childhood residence, apparent geographical differences in SLE by census region or latitude (a higher prevalence of SLE cases than non-cases in the South and lower latitude) were non-significant after adjusting for race/ethnicity (Table 3). Odds of SLE were somewhat elevated for living in a rural compared with a suburban environment; however, when compared with all other areas combined, the association of SLE with rural residence was significant (OR = 1.6; 95%CI 1.1, 2.3). An association was seen for SLE with use of residential pesticides in childhood, with a dose response for more frequent use (none, less than once per month, or at least once per month; *p*-trend < 0.0001). This association was not confounded by adult residential pesticide use (reported for current or longest held adult residence by 55% of cases and 45% of non-cases), even though use of childhood residential pesticides was more common in women who reported adult residential pesticides (not shown). Compared to women who did not report any residential pesticide use, SLE was significantly associated with having used pesticides in both childhood and as an adult (OR = 1.8; 95%CI 1.1, 3.1) and a non-significant association of similar magnitude was seen for childhood-only use (OR = 1.6; 95%CI 0.88, 2.9) (Table S2 in Supplementary Material), supporting an independent association with childhood-pesticide use regardless of adult use.

**Table 3:** **Characteristics of longest childhood residence and associations with SLE**.

	SLE		Odds ratios^b^ (95% confidence intervals)^a^

	Probable *N* = 119	Non-case *N* = 48,950

	*N* (%)	*N* (%)	
**Longest childhood residence^a^**			
U.S. Region			
West	18 (15)	7,032 (14)	1.3 (0.70, 2.3)
Midwest	32 (27)	17,443 (36)	Referent
Northeast	30 (24)	12,087 (25)	1.4 (0.83, 2.2)
South	36 (29)	11,428 (23)	1.4 (0.85, 2.3)
Puerto Rico	3 (3)	915 (2)	NC
Latitude			
≤35°	29 (24)	7,918 (16)	Referent
35–40°	30 (25)	11,461 (12)	0.90 (0.52, 1.5)
>40°	60 (50)	29,526 (60)	0.75 (0.46, 1.2)
Environment			
Urban	26 (21)	11,324 (23)	0.83 (0.49, 1.4)
Suburban/other	33 (27)	13,927 (27)	Referent
Small town	24 (20)	11,663 (23)	0.79 (0.47, 1.3)
Rural	39 (32)	12,851 (20)	1.4 (0.86, 2.0)
Residential pesticides^c^			
Never used	70 (61)	34,500 (79)	Referent
Infrequent (<monthly)	29 (25)	7,657 (17)	1.6 (1.0, 2.5)
Frequent (monthly+)	15 (13)	2,534 (6)	2.3 (1.3, 4.1)
Residence			
Was not a farm	67 (58)	28,960 (64)	Referent
Near a farm	15 (13)	6,213 (14)	1.1 (0.65, 2.6)
Used to be a farm	10 (9)	2,971 (7)	1.6 (0.80, 3.0)
Was a farm	24 (21)	7,093 (16)	1.6 (1.0, 2.6)^c^
**Early life and extended childhood farm residence^d^**			
No maternal or childhood	81 (74)	33,272 (77)	Referent
Only maternal/prenatal	6 (5)	3,293 (8)	0.70 (0.30, 1.6)
Only childhood	3 (3)	1,869 (4)	NC
Both maternal and childhood	20 (18)	4,963 (11)	1.8 (1.1, 3.0)

*^a^Residence location was missing or outside the U.S. for 5 cases and 1,560 non-cases. Women were grouped by state into census regions and by latitude based on majority of state area and/or population*.

*^b^Odds ratios calculated by logistic regression, adjusted for age and race/ethnicity; not calculated (NC) if 3 or fewer cases*.

*^c^Use by self or others; *p*-trend = 0.002*.

*^d^Mother lived or worked on a farm while pregnant and longest childhood residence was a farm; referent group includes women who lived near a farm or on a former farm and women with other short-term farm experiences*.

While having a long-term childhood farm residence was associated with SLE, when coupled with information on maternal farm history, this reflected the association with an early and extended farm experience (i.e., prenatal farm exposure and longest childhood farm residence was a farm OR = 1.8; 95%CI 1.1, 3.0) (Table 3). Few cases (*n* = 3) reported having their longest childhood residence on a farm in the absence of maternal/prenatal farm experience, and maternal/prenatal farm exposure alone was not associated with SLE. Based on data from the more detailed farm residence questionnaire, SLE was associated with living on a farm at least 12 months in childhood-only, but not as an adult (Table 4). In this context, SLE was associated with self-reported personal exposure to pesticides in childhood through application to crops. An elevated, non-significant association was seen for use on animals, and a stronger association was seen for exposure to pesticides from both animals and crops compared to either one alone. For those women with data on both longest childhood farm residence and agricultural pesticide use, the majority (80%) of cases and (70%) non-cases who reported agricultural pesticides also had an early and extended farm residence (from Table 3), so independent effects could not be assessed.

**Table 4:** **Farm residence and agricultural pesticide use associated with SLE**.

	SLE		Odds ratios^a^ (95% confidence intervals)
	Probable	Non-case	
		
	*N* = 124 *N* (%)	*N* = 50,465 *N* (%)	
**Lived on farm 12+ months**			
Never	87 (72)	37,288 (77)	Referent
Childhood only	28 (23)	7,348 (15)	1.7 (1.1, 2.7)
Child and adulthood	4 (3)	2,275 (5)	0.85 (0.31, 2.3)
Adult only	2 (2)	1,757 (4)	0.57 (0.14, 2.3)
**Childhood-only farm pesticide exposures versus never lived on farm**			
Never lived on farm	87 (76)	37,288 (85)	Referent
Farmed – pesticides on crops			
None used	8 (7)	3,132 (7)	1.2 (0.58, 2.5)
No personal exposures	6 (5)	2,479 (6)	1.1 (0.47, 2.5)
Personal exposures	13 (11)	1,288 (3)	4.2 (2.4, 7.7)
Farmed – animals			
No livestock contact	7 (6)	2,184 (5)	1.4 (0.66, 2.9)
Contact, no pesticides used	13 (11)	3,676 (8)	1.5 (0.90, 2.6)
Contact, pesticides used	7 (6)	1,118 (3)	2.1 (0.98, 4.6)
Personal pesticide exposures			
None	7 (6)	2,744 (6)	1.2 (0.55, 2.6)
Either crops or livestock	14 (12)	3,233 (7)	1.9 (1.1, 3.3)
Both crops and livestock	6 (5)	826 (2)	3.5 (1.5, 8.2)

*^a^Odds ratios calculated by logistic regression, adjusted for age and race/ethnicity; not calculated (NC) if 3 or fewer cases*.

## Discussion

These findings support the idea that perinatal and early life exposures may influence risk of developing SLE. Ours is the first study to report SLE associations with more frequent use of pesticides at the childhood residence and with maternal/prenatal and childhood farm residence. We also saw that SLE was associated with personal exposure to agricultural pesticides in women who had lived or worked on a farm for 12 months or more up to age 18 in the absence of adult farm exposures. Together, these results suggest a dose-related elevation in SLE risk associated with early life pesticide exposure. Prior studies have suggested associations of SLE with farming occupation or personal pesticide exposures SLE (18–21), but the relevant timing of exposures was not known. These novel findings warrant replication in other populations.

We also saw associations of SLE with low birthweight and preterm birth. The independent effects of preterm birth may be difficult to identify due to the close relationship between these two factors, however, the birthweight association remained consistent when excluding preterm births. Prior evidence on birthweight and SLE is inconsistent, with one study showing SLE associated with high birthweight in women (12) and null findings in other studies (13, 24). Reasons for an apparent inconsistency across studies are not clear. In the prior study (combining two cohorts of the Nurses’ Health Study, NHS) (12), the association with high birthweight was most apparent for the older cohort (mean age of 46 in 1976), whereas the younger cohort (mean age of 35 in 1989) showed only modest and non-significant associations of SLE with both high (OR = 1.3) and low (OR = 1.5) birthweight. The current study participants were more similar to the younger NHS cohort; however, cases in the NHS cohorts were incident, whereas the current study case sample included prevalent cases and could be biased if higher birthweight was related SLE severity or other reasons for non-participation. In the most recent study, linking registry data on births and SLE diagnoses in Sweden (13), the population and cases were considerably younger (average age at onset of 21 years). However, this study did not rely on recall for birthweight and had a sufficient sample size to adjust for gestational age.

Prematurity has been associated with SLE in females and younger male cases (12, 13). In the present study sample, a similar proportion of cases and non-cases were missing data (compared with term birth OR = 1.1; 95%CI 0.71, 1.6 for “missingness” adjusted for age and race/ethnicity), so we did not impute missing values. Under the assumption that preterm birth is an event that is well reported if it occurs, we performed a sensitivity analysis assigning missing values to “term” birth, and results were unchanged. Many pathways and mechanisms might link birthweight and prematurity to increased risk of SLE (6, 25, 26). Limitations in the present study sample size and data precluded further investigations of life-course pathways, possibly operating through other developmental factors, such as age at menarche or adult obesity (27).

We saw no significant associations with social factors when race/ethnicity was included in the model, which is different from our previous findings in RA (22). Minority race/ethnicity may contribute to social disparities, and associations with young maternal age and SES score were statistically significant when only adjusted for age (not shown). We also noted that childhood SES score appeared to be associated with SLE regardless of adult educational attainment (Table S1 in Supplementary Material). Lower adult education was also associated with SLE, but we did not see any stronger association for women with both low adult education and two or more childhood SES factors as we did for RA (22). We cannot rule out the influence of selection bias. Lower SES has been associated with higher morbidity and mortality in SLE (14, 28, 29), so one possibility is that SLE cases with, long-term lower SES and greater health disparities may have been unable to enroll in the cohort.

Few other perinatal factors were associated with SLE, including maternal or household smoking, birth season, breast feeding, being first born or number of siblings. Geographic differences in SLE across the U.S. have been previously reported, but are difficult to disentangle from racial differences (30, 31). In our analyses, higher latitude (proxy for lower UVB) was inversely associated with SLE risk in age-adjusted models (not shown), which was attenuated after adjusting for race/ethnicity.

We had no data on specific pesticides or types of pests treated. Organochlorines such as DDT have previously been linked to SLE risk (1). A causal association of organochlorine insecticides and SLE in susceptible individuals is supported by experimental studies, in which treatment with chlordecone, methoxychlor, or *o*,*p′*-DDT accelerated SLE disease onset, including earlier increases in autoantibody levels and renal impairment in lupus-prone mice, but not in a non-susceptible mouse strain. Many other pesticides impact the immune system through diverse mechanisms (32) and bear further investigation in experimental models also taking into account the question of timing relative to developmental windows of susceptibility.

Our study has several limitations, one being the use of self-reported cases. Validation studies are costly, however, and can reduce sample size and generalizability due to biased participation in studies requiring release of medical information. Because self-report is notoriously non-specific for lupus, we increased specificity of our case definition by confirming probably clinical cases based on the self-reported use of DMARDs (33). This definition does not identify all cases, including those with incomplete medication histories or those receiving non-DMARD treatments. However, a prevalence of ~2 cases per 1,000 (e.g., 124/50,000) is reasonable for this cohort, given an estimated population prevalence of 1/1,000, with higher rates in women. If socioeconomic factors and other environmental exposures are related to severity and treatment with DMARDs, then our case definition may also lead to underestimated associations with disease. Disease severity may influence treatments, survival or participation in the cohort, limiting generalization of these findings to SLE in the broader population.

These analyses relied on self-reported data on perinatal and childhood factors. Sensitivity analyses were conducted, limiting the sample to women under age 60, as reporting accuracy is expected to diminish with age and years since exposure, and younger women may be more likely to be able to consult their mothers for assistance. In most instances, we did not see changes in associations. Difficulty in reporting birthweight and preterm birth was obvious, as evidenced by the high number of missing data. Other factors may be more easily and objectively reported, such as childhood farm residence. Some immune modifying factors were not considered because they were rare (e.g., pregnancy-related hypertensive disorder or DES exposure) or not assessed (e.g., maternal nutrition) (34). Reporting of childhood pesticides may be influenced by adult pesticide use, which in turn may be influenced by health status.

Strengths of the study include the broad range of early life data available in the cohort, and results showing internally consistent findings for residential and agricultural pesticides and birthweight/prematurity. Replication of these analyses using incident cases, using a life-course approach, is warranted.

## Author Contributions

CP conceived of the topic, obtained, and managed the data, designed and conducted the analyses, interpreted findings, and wrote the paper. AD and DS contributed expertise on exposures, obtained and manage data, assisted with analysis design and interpreted findings, and critically reviewed the paper prior to submission. All authors agreed to final draft and submission.

## Conflict of Interest Statement

The authors declare that the research was conducted in the absence of any commercial or financial relationships that could be construed as a potential conflict of interest.
