# Supplementary material for: Early Life Factors Associated with Adult-Onset Systemic Lupus Erythematosus in Women
Source: Front Immunol. 2016 Mar 31;7:103. doi: 10.3389/fimmu.2016.00103 (PMC4814765; doi:10.3389/fimmu.2016.00103)
Supplement: Supplementary file 1 [file Table_1.PDF]

Supplemental Table 1 Adult characteristics	Childhood characteristics	Cases N= 118	Non-cases N=48,388	Odds Ratio (95%CI) Age, race-adjusted[1]
Educational attainment	*SES Factors*	%	%	
≥ College degree	None or one	24	40	Referent
	Two or more	15	11	2.0 (1.2, 3.6)
< College degree	None or one	40	31	2.1 (1.4, 3.4)
	Two or more	20	18	1.8 (1.0, 3.0)

[1]Odds ratios calculated by logistic regression

[2]Missing data for childhood SES factors on 6 cases and 2073, and adult education on an additional 6 non-cases
