# Supplementary material for: Early Life Factors Associated with Adult-Onset Systemic Lupus Erythematosus in Women
Source: Front Immunol. 2016 Mar 31;7:103. doi: 10.3389/fimmu.2016.00103 (PMC4814765; doi:10.3389/fimmu.2016.00103)
Supplement: Supplementary file 2 [file Table_2.PDF]

Supplemental Table 2

Residential pesticide use	Cases N= 144	Non-cases N=44,904	Odds Ratio (95%CI) Age, race-adjusted[1]
	%	%	
None	38	48	Referent
Childhood only	13	9	1.6 (0.88, 2.9)
Adult only	23	29	0.96 (0.53, 1.6)
Both	26	14	1.8 (1.1, 3.1)

[1]Odds ratios calculated by logistic regression

[2]Missing data for childhood pesticide use on 9 cases and 5237 non-cases, and current pesticide use on an additional 1 case and 312 non-cases
